# Supplementary material for: ProfPPIdb: Pairs of physical protein-protein interactions predicted for entire proteomes
Source: PLoS One. 2018 Jul 18;13(7):e0199988. doi: 10.1371/journal.pone.0199988 (PMC6051629; doi:10.1371/journal.pone.0199988)
Supplement: S1 Appendix — Detailed results from training and testing the PPIs. (PDF) [file pone.0199988.s001.pdf]

S1 Appendix

ProfPPIdb: pairs of physical protein-protein interactions predicted for entire proteomes

Linh Tran[1,2,*], Tobias Hamp[2], Burkhard Rost[2,3],

1 Imperial College London (ICL), Department of Computing, London SW7 2AZ, United Kingdom
2 Technical University of Munich (TUM), Department of Informatics, Bioinformatics & Computational Biology - i12, Boltzmannstr. 3, 85748 Garching, Germany
3 Technical University of Munich (TUM), Institute for Advanced Study (TUM-IAS), Lichtenbergstr. 2a, 85748 Garching, Germany

* linh.tran@imperial.ac.uk

A Cross validation results

Fig A. Cross-validation and holdout performance results for organisms with train data size > 500 PPIs. The y-axes give precision (number of PPIs correctly predicted at threshold), the x-axes the recall (number of experimental interactions predicted at that threshold). Bars give the standard deviation; negatives were sampled at a rate of 10:1 (ten negatives for one positive). Each subplot is referred as follows: A (*Escherichia coli)*, B (*Caenorhabditis elegans)*, C (*Drosophila melanogaster)*, D (*Arabidopsis thaliana)*, E (*Mus musculus)*.

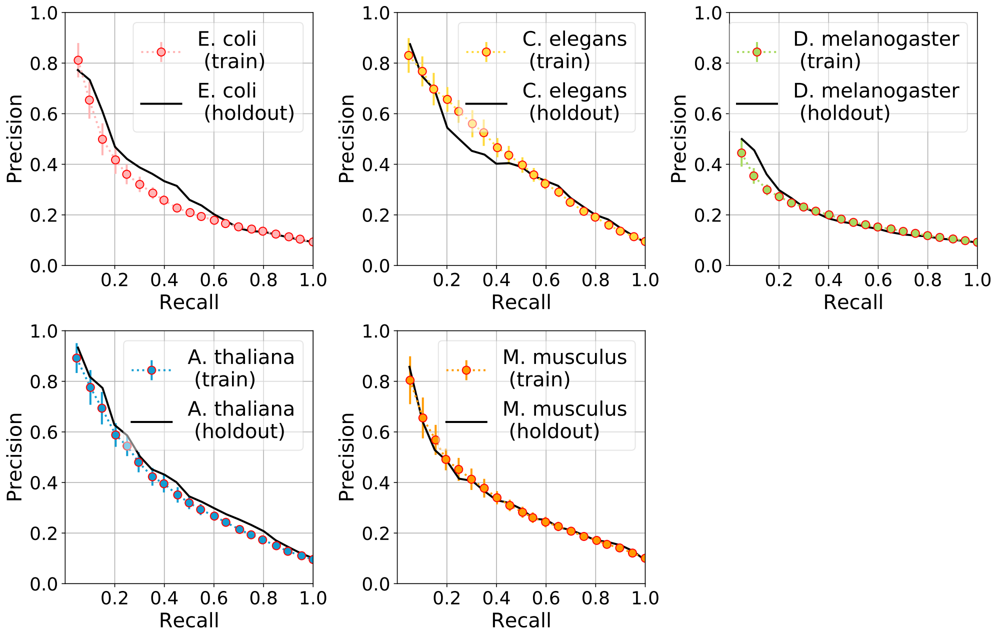

Similar levels of training and holdout performances Machine learning applications often reach very different levels of performance for the training and the

Fig B. Cross-validation and hold-out performance results of *Escherichia coli*. **Panel (A)**: Precision-recall curve for cross-validation in *Escherichia coli* with different optimization hyperparameters. All results in the paper were reported for the version $k = 5/\sigma = 11$ which clearly was not best for *Escherichia coli,* instead the combination $k = 3/\sigma = 4$ yielded the best performance (purple). **Panel (B)**: Comparison of cross-validation hyperparameter combinations $k = 3/\sigma = 4$ (best) with $k = 5/\sigma = 11$ (default) and cross validation of human from earlier publication [1]. **Panel (C)**: Cross-validation and hold-out results of hyperparameter combination $k = 3/\sigma = 4$ (best) compared with test results for human [1].

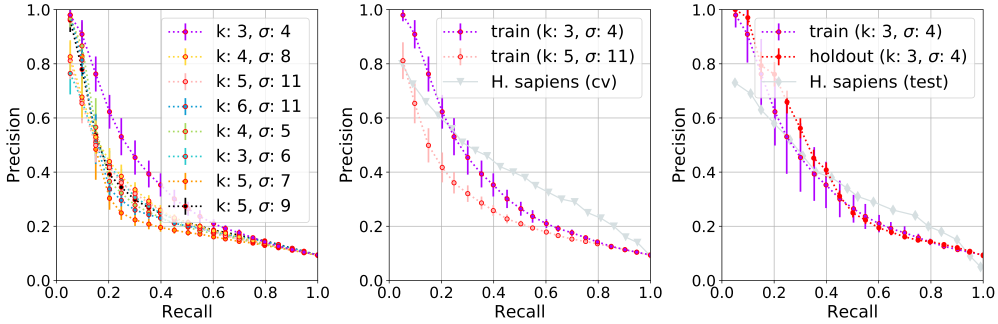

testing set. We did not observe this for the organisms for which we could compile comprehensive cross-validation results (Fig. A: difference between black line and colored points). Most similar were the results for mouse (*Mus musculus:* Fig. A E). For *Escherichia coli* (Fig. A A), *Caenorhabditis elegans* (worm, Fig. A B), and *Drosophila melanogaster* (fruit fly, Fig. A C), training and testing were less similar for high recall, i.e. for the most reliable predictions. Most unusual were the results for *Drosophila melanogaster* (Fig. A C) and *Escherichia coli* (Fig. A A), for which test performance was even higher than training performance for a substantial fraction of highly reliable predictions (toward left, i.e. low recall in Fig. A A, and Fig. A C the black curves are above the dots). For *Arabidopsis thaliana* (water-cress, Fig. A D) testing performance was better than training throughout the entire ROC-like curve. Typically, there is only one explanation for such unexpected findings: points for which testing is better than training provide estimates for the resolution of our performance estimates. This reality was captured well by the estimates for standard errors: within one standard error, training and testing were identical for all organisms.

Hyperparameter optimization for *Escherichia coli* Our most important objective when applying machine learning typically is to reduce the risk of over-optimization, i.e. to optimize generalization instead of apparent performance as usually over-estimated by standard cross-validation. Therefore, we trained each organism model with the same set of hyperparameters (k-mer = 5 and $\sigma = 11$). This standard choice yielded the best performance for almost all organisms. One exception was Escherichia coli. For the choice k-mer = 3 and $\sigma = 4$, the cross-validation precision-recall values exceeded those for all other hyperparameter combinations (Fig. B A). This top choice for *Escherichia coli* reached higher performance than the human-specific model in the realm of low recall (Fig. B B). This choice for *Escherichia coli* also results in high performance for the holdout set of *Escherichia coli* which exceeds the test performance of *Homo sapiens* from [1] especially in the realm of low recall (Fig. BC).

Table 1. Summary of experimental evidences found in BioGRID [2], DIP [3], and IntAct [4]. Organism: latin name for eight model organisms sorted alphabetically; NpredPPIs:Number of PPIs of 1% ranked predictions; NEvidence: number of PPIs for which experimental evidences was found in at least on of the three databases used for training; NcorrectEvidence: number of PPIs with experimental evidence which were correctly classified by our approach; Accuracy: The fraction of correct predictions within the predictions with experimental evidence.

Organism	NpredPPIs	NEvidence	NcorrectEvidence	Accuracy
A. thaliana	2,064,410	62	60	96.77 %
C. elegans	1,421,719	67	69	97.1 %
D. melanogaster	319,160	152	197	77.16 %
E. coli	27,296	82	90	91.11%
M. musculus	1,313,253	0	0	-
P. falciparum	90,418	143	174	82.18%
S. pombe	83,497	166	177	93.79%
R. norvegicus	2,119,225	3	0	0.00
Sum over all 8	6,125,724	772	670	86.79 %

B Evaluation of novel predictions

We used **BioGRID** [2], **DIP** [3], and **IntAct** [4] (Uniprot uses quality-filtered subset of binary interactions automatically derived from the IntAct database) for large-scale evaluation of our novel predictions. Although we used BioGRID [2], DIP [3], and IntAct [4] as the base for our organism-specific models, it was only a small subset of the databases' PPIs used for training our models.

Fig C. Percentages of predictions as a function of PPI quality score according to expert knowledge scoring scheme [5]. This scoring scheme was also used in the manuscript to obtain high-quality PPIs for training. The positive and negative PPIs presented in these plots are findings of experimental evidences found in BioGRID [2], DIP [3], and IntAct [4].

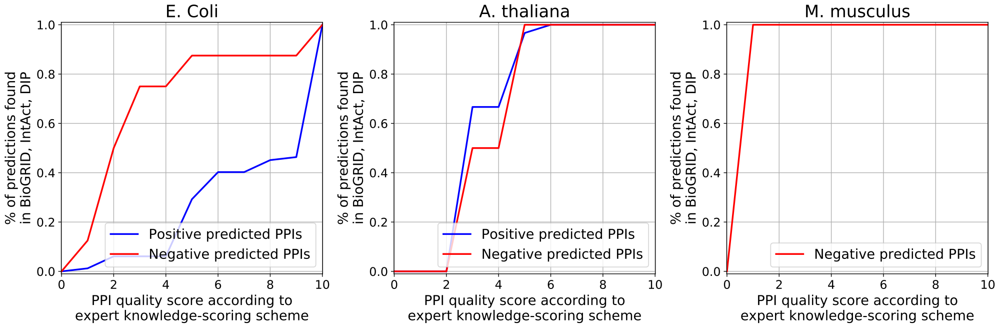

The PPIs published on our online service only include PPIs which have not any experimental evidence from any of these three databases. In order to perform an evaluation of the quality of the predictions, we used the top 1 % of all predictions (ranked according to our confidence measure) which were not included in the training set. We compared these top predictions against all experimental from BioGRID [2], DIP [3], and IntAct [4]. The findings of experimental evidences is listed in Table 1. As Table 1 shows, except for *Mus musculus* and *Rattus norvegicus* for which none or only falsely predicted PPIs was found, we found between 60 and 170 PPIs with experimental

evidence for each organism. The accuracy of the evidences correctly predicted is at least over 75 %, with half of all investigated organisms having accuracies of over 90 %.

Looking closer at the distribution of the evidences in terms of average, we found three cases which we show in Figure C. With *Escherichia coli* (Figure C a), we observe a high percentage of lower average expert knowledge scores (below 4 for almost 80 % of the evidences) for negative PPIs, and a high percentage of high average expert knowlege scores (greater or equal 6 for 60 % of the evidences found). This shows that for *Escherichia coli* our model succeeds in predicting PPIs correctly which also has experimental evidences with high average expert knowledge scores. However, for organisms *Arabidopsis thaliana* (Figure C b), *Caenorhabditis elegans, Drosophila melanogaster, Plasmodium falciparum* and *Schizosaccharomyces pombe* we witness PPI curves which almost overlaps. This indicate a similar distribution of knowledge expert scores for both positive and negative PPIs. This also is a consequence of the lack of high quality annotations present in the databases. The third case of distribution that we observed is with *Mus musculus,* for which we only found three experimental evidences and none of them are correctly predicted by our approach. Nevertheless, the experimental evidences are highly doubtful as their methods each score only 1 in the expert knowledge scoring system.

References

1. Hamp T, Rost B. Evolutionary profiles improve protein–protein interaction prediction from sequence. Bioinformatics. 2015;31(12):1945–1950.

2. Stark C, Breitkreutz BJ, Reguly T, Boucher L, Breitkreutz A, Tyers M. BioGRID: a general repository for interaction datasets. Nucleic acids research. 2006;34(suppl 1):D535–D539.

3. Xenarios I, Salwinski L, Duan XJ, Higney P, Kim SM, Eisenberg D. DIP, the Database of Interacting Proteins: a research tool for studying cellular networks of protein interactions. Nucleic acids research. 2002;30(1):303–305.

4. Kerrien S, Alam-Faruque Y, Aranda B, Bancarz I, Bridge A, Derow C, et al. IntAct—open source resource for molecular interaction data. Nucleic acids research. 2007;35(suppl 1):D561–D565.

5. Schaefer MH, Fontaine JF, Vinayagam A, Porras P, Wanker EE, Andrade-Navarro MA. HIPPIE: Integrating protein interaction networks with experiment based quality scores. PloS one. 2012;7(2):e31826.
