# Supplementary figures and images for: ProfPPIdb: Pairs of physical protein-protein interactions predicted for entire proteomes
Source: PLoS One. 2018 Jul 18;13(7):e0199988. doi: 10.1371/journal.pone.0199988 (PMC6051629; doi:10.1371/journal.pone.0199988)

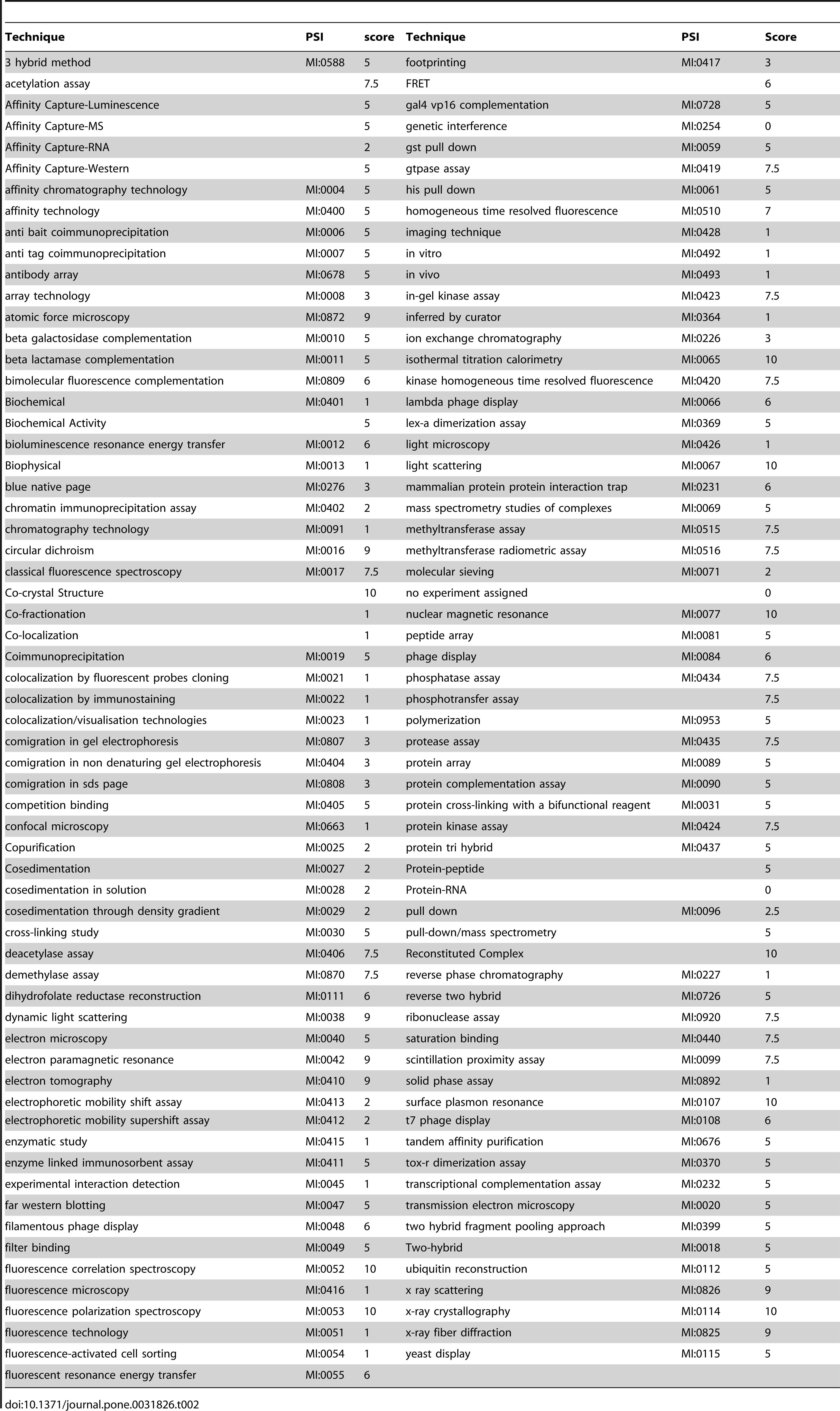

Supplement: S4 Fig — These scores were used for selection of high-quality (reliable) PPIs. (TIF) [file pone.0199988.s005.TIF]
